# Generative adversarial network–based reconstruction of healthy anatomy for anomaly detection in brain CT scans

**DOI:** 10.1117/1.JMI.11.4.044508

**Published:** 2024-08-09

**Authors:** Sina Walluscheck, Annika Gerken, Ivana Galinovic, Kersten Villringer, Jochen B. Fiebach, Jan Klein, Stefan Heldmann

**Affiliations:** a Fraunhofer Institute for Digital Medicine MEVIS, Lübeck, Germany; b Universitätsmedizin Berlin, Center for Stroke Research Berlin (CSB) Charité, Berlin, Germany

**Keywords:** head, deep learning, detection, anomaly, brain, computed tomography

## Abstract

**Purpose:**

To help radiologists examine the growing number of computed tomography (CT) scans, automatic anomaly detection is an ongoing focus of medical imaging research. Radiologists must analyze a CT scan by searching for any deviation from normal healthy anatomy. We propose an approach to detecting abnormalities in axial 2D CT slice images of the brain. Although much research has been done on detecting abnormalities in magnetic resonance images of the brain, there is little work on CT scans, where abnormalities are more difficult to detect due to the low image contrast that must be represented by the model used.

**Approach:**

We use a generative adversarial network (GAN) to learn normal brain anatomy in the first step and compare two approaches to image reconstruction: training an encoder in the second step and using iterative optimization during inference. Then, we analyze the differences from the original scan to detect and localize anomalies in the brain.

**Results:**

Our approach can reconstruct healthy anatomy with good image contrast for brain CT scans. We obtain median Dice scores of 0.71 on our hemorrhage test data and 0.43 on our test set with additional tumor images from publicly available data sources. We also compare our models to a state-of-the-art autoencoder and a diffusion model and obtain qualitatively more accurate reconstructions.

**Conclusions:**

Without defining anomalies during training, a GAN-based network was used to learn healthy anatomy for brain CT scans. Notably, our approach is not limited to the localization of hemorrhages and tumors and could thus be used to detect structural anatomical changes and other lesions.

## 1 Introduction

In recent years, the number of computed tomography (CT) examinations has increased significantly, resulting in a high workload for radiologists. The resulting time pressure increases the risk of reduced quality and safety of diagnoses and radiologist burnout.^1^ Therefore, artificial intelligence solutions have been developed to address this issue and provide quality diagnosis as a second opinion or pre-diagnosis.

In this work, we deal with CT brain examinations and aim to support radiologists in finding relevant pathologies, such as hemorrhages, tumors, or ischemia.

Various methods have been proposed to assist radiologists in this task. In particular, deep learning-based approaches have shown very good results in many applications and are now the technology of choice. Deep learning methods for automated anomaly detection can generally be divided into two types.

The first type is designed to find specific pathologies. Therefore, a model is trained to detect or segment a particular type of abnormality, usually with a supervised or semi-supervised approach using annotated training data.^2^ Good detection performance of such methods has been demonstrated in the literature, but they are inherently limited to the specific types of findings for which they have been trained, and at best, unknown abnormalities are detected as false positives. The second type of method takes a fundamentally different approach. They do not make any assumptions and do not try to find a specific pathology but are designed to detect any deviation from “normal.” Therefore, training such models does not require reference segmentations of pathologies but is usually based on (unsupervised) learning a representation of normal anatomy. Thus, it potentially has fewer limitations, and a trained model should be less error-prone for unseen anomalies.

Due to that advantage, we follow the latter approach for our method of general anomaly detection in brain CT images.

Architectures used for this type of anomaly detection include generative adversarial networks (GANs), variational autoencoders (VAE), autoencoders (AE), and combinations of these methods.^3^^–^^6^ Reconstruction of a normal image is then generally done by mapping an input image with an encoding network to a latent space and reconstructing the output image with a decoding network. In the so-called restoration approaches, the encoding is not performed by a trained network but through iterative optimization with respect to the input in the latent space of the trained decoding network, such that the decoded (or restored) output image is most similar to the given input image. Baur et al.^7^ compared the different unsupervised approaches for magnetic resonance imaging (MRI) brain images and found that a VAE with restoration and the “f-AnoGAN,”^8^ originally proposed for optical coherence tomography data and combining a Wasserstein GAN^9^ with encoder training, performed best. In addition, diffusion models have recently been investigated for anomaly detection. Wyatt et al.^10^ obtained Dice values of around 0.38 on MRI images with brain tumors. Much research has been done on MRI images, but simply using these models as is does not solve the problem of detecting abnormalities in CT images of the brain. The imaging properties of CT imply a high contrast between the skull and the brain tissue with much more subtle gray versus white matter differences. Thus, the generated reconstructions must also reflect these properties while especially preserving visible details inside the brain with lower grayscale contrast.

For brain CT data, Viana et al.^11^ utilized a 3D implementation of the f-AnoGAN to detect traumatic brain injuries. Toikkanen et al.^12^ developed a method to segment intracranial hemorrhages in brain CT images and combined a GAN with an encoder to reconstruct pseudo-normal versions of abnormal input images. Their reconstructions however do not correct structural changes of the brain, such as ventricle deformations, that might occur due to hemorrhages. They achieve Dice scores of around 0.7 while focusing on the segmentation of hemorrhages only. In a recent publication, Lee et al.^13^ described a combination of GAN and encoder training for CT emergency triage. They collected a large dataset of more than 34,000 patients for training and used iterative latent code and noise optimization during inference, showing very promising results.

In summary, there is very little work on unsupervised detection of brain anomalies in CT. Autoencoders often tend to produce blurry results that lack detail. Diffusion models are expected to result in higher image quality than VAEs and require less training data than GAN approaches. On the other hand, reconstruction times are very long, and high image resolutions, which have been shown to be beneficial for anomaly detection,^4^ might be hard to obtain due to computational requirements. Furthermore, when using diffusion models to reconstruct normal versions of an image, the choice of noise distribution used is critical in determining what sizes of anomalies can be recovered (see Wyatt et al.^10^).

The contribution of this work is that we propose an efficient unsupervised GAN-based method to localize different types of lesions in CT scans of the brain. We choose a GAN-based architecture over others because of the above-mentioned disadvantages. We aim to generate high-resolution healthy image reconstructions and want to ensure that a representation of normal anatomy is learned first so that our reconstructions will not contain abnormalities. In this work, we show that with the right choice of GAN training, we can achieve high-quality reconstruction results even on a moderate-sized training dataset. The paper is structured by first describing the GAN training and our two image reconstruction approaches, followed by the generation of anomaly segmentation maps and an evaluation of CT images with hemorrhages and tumors. We also compare our method with two non-GAN-based approaches.

## 2 Material and Methods

### 2.1 Data

We use the publicly available challenge data for intracranial hemorrhage detection provided by the Radiological Society of North America (RSNA).^14^ Originally, this dataset contains more than 25,000 CT head examinations with slice-wise annotations for the type of hemorrhage present, collected at various sites in California, Philadelphia, and Brazil using scanners from different manufacturers. The resolution of the images in the axial plane ranges from 0.4 to 0.6 mm with a slice thickness of 5 mm. In addition to the annotations provided, two of our radiologists (I.G. and K.V.) with more than 10 years of experience in neurological imaging also annotated a subset of these data by marking slices without findings as “normal.” The RSNA annotations did not include such information but only hemorrhage annotations. We export more than 1000 normal 2D slices with a size of 512×512  pixels. We also select a subset of 30 images with hemorrhages for testing, where our radiologists have delineated the hemorrhages present. We restrict both subsets to mid-axial slices of the brain to limit the complexity of the problem in the first step. The number of slices that can be used from the RSNA dataset is reduced because of the limited annotation time and because we only use center slices of the brain. The image slices were grouped according to patients. Images from one patient are only used for either training or testing.

For testing, we also include tumor data from the “Glioma Image Segmentation for Radiotherapy” study from the Cancer Imaging Archive^15^ and extract 23 slices that have been annotated by one of our radiologists. We therefore obtain reference anomaly masks for an independent dataset.

Thus, in total, we used 1180 “normal” 2D axial images to train our GAN. In addition, we obtain a total of 53 segmented images with pathologies (30 with hemorrhages and 23 with tumors). Subsequently, we determined an operating point for the anomaly detection on a validation set of 27 axial images with segmented pathologies (15 scans with hemorrhages and 12 scans with tumors). Then, we tested our method on 26 test images (15 scans with hemorrhages and 11 scans with tumors). A summary of the data split for testing and validation is shown in Table 1.

**Table 1:** Composition of validation and test dataset with pathologies. A total of 53 images were manually segmented by two radiologists. The images show hemorrhages or tumors and were split into test and validation sets as shown.

	Hemorrhages	Tumors
Validation	15	12
Test	15	11
Total	30	23

### 2.2 Architecture and Training

**Fig. 1:**
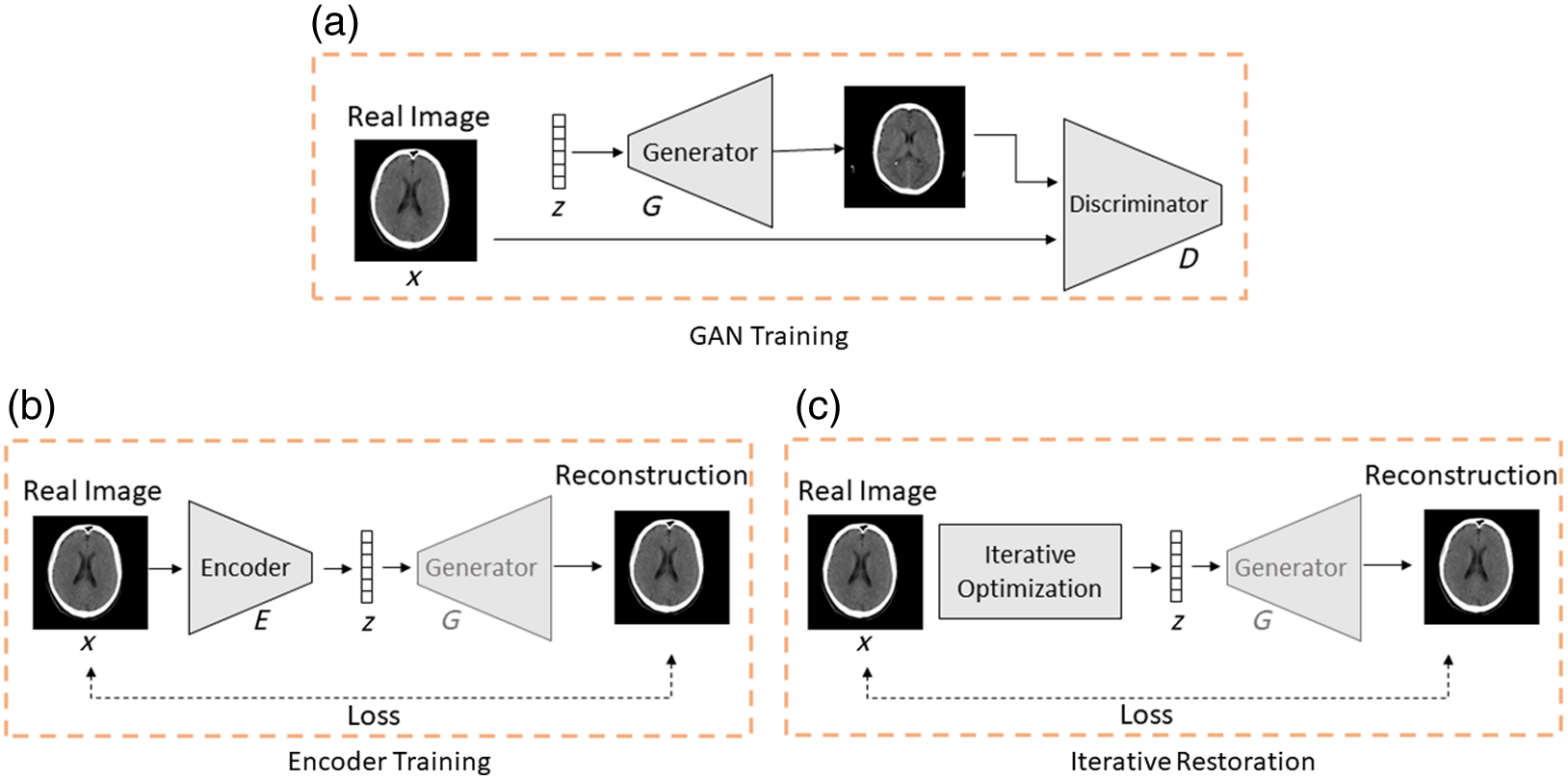
Overview of our network training and image restoration. (a) Step 1: training the generator and discriminator of a GAN with real healthy images. (b) Step 2: training an encoder with a fixed generator. (c) Iterative optimization of z during inference.

The architecture of our network is schematically shown in Fig. 1. To ensure learning a representation of normal brain anatomy, we decided to combine a GAN architecture with an image encoding step (using an encoder network or an iterative optimization). Thus, we can later compare two different encoding approaches. As shown in Fig. 1, we have two subsequent training steps: First, the GAN is trained to generate normal CT slices from a latent space vector [see Fig. 1(a)], and second, the encoder is trained to map healthy input images to the latent space [see Fig. 1(b)]. During inference, an input CT is mapped with the encoder to the latent space and from there back to the image space with the generator.

As an alternative that requires no training of an encoder network, we implement a restoration approach. Here, we iteratively optimize over the latent space to find an optimal input vector for the decoder so that a given image is reconstructed in the best possible way [see Fig. 1(c)]. We describe all steps in detail in Secs. 2.2.1–2.2.4.

#### 2.2.1 GAN training: generating normal CT images

The first part of our method is to train a GAN to learn representations of normal CT images of the brain. Based on literature research, we use a network architecture after Liu et al.,^16^ which was proposed for non-medical images and has been shown to be capable of generating high-resolution images from even small training sets. Also, we verified in our own initial tests that this architecture leads to better results compared with a Wasserstein GAN^9^ architecture. The code base for the discriminator and generator can be found at https://github.com/odegeasslbc/FastGAN-pytorch/tree/main. We use the proposed architecture with noise injection, spectral normalization,^17^ and image augmentation (random mirroring, contrast changes, and translation). Only a small change was made to the architecture as we use smaller feature maps (four instead of eight) as input to the decoder that is part of the discriminator. In addition, we leave one generator upsampling block out as we only work with an image size of 512. The model is trained with a batch size of 8 for 100,000 steps, using the learning rate of 2e−4 and Adam optimizer.

For our experiments, we used a training dataset consisting of 1180 brain CT slices. Our code is written in PyTorch, and we train on an NVIDIA GeForce RTX 2080 Ti GPU.

In the first step, we alternatingly train the generator G and the discriminator D (see Liu et al.^16^ for model implementation details). This is done by minimizing the generator loss

$$L_{G}=-E_{z∼U[-1,1]}[D(G(z))]$$ (1)

and the discriminator loss

$$L_{D}=E_{x∼I_{\text{real}}}[\max(0,1-D(x)]+E_{z∼U[-1,1]}\;\max(0,1+D(G(z))]+L_{\mathrm{rec}},$$ (2)

where $I_{\text{real}}$ denotes the distribution of real normal CT scans, z∼U[−1,1] are latent vectors with components uniformly distributed over the interval [−1,1], and $L_{\mathrm{rec}}$ is an additional reconstruction loss that originally was introduced by Liu et al.^16^ to enforce the extraction of meaningful image features in the discriminator (for details, see Ref. 16). Here, we use the reconstruction loss $L_{\mathrm{rec}}$ as proposed in Ref. 18.

After training, we can use the generator to obtain normal brain images from a random uniformly distributed latent vector $z\in {\left[-1,1\right]}^{512}$.

#### 2.2.2 Image reconstruction: encoder training

**Fig. 2:**
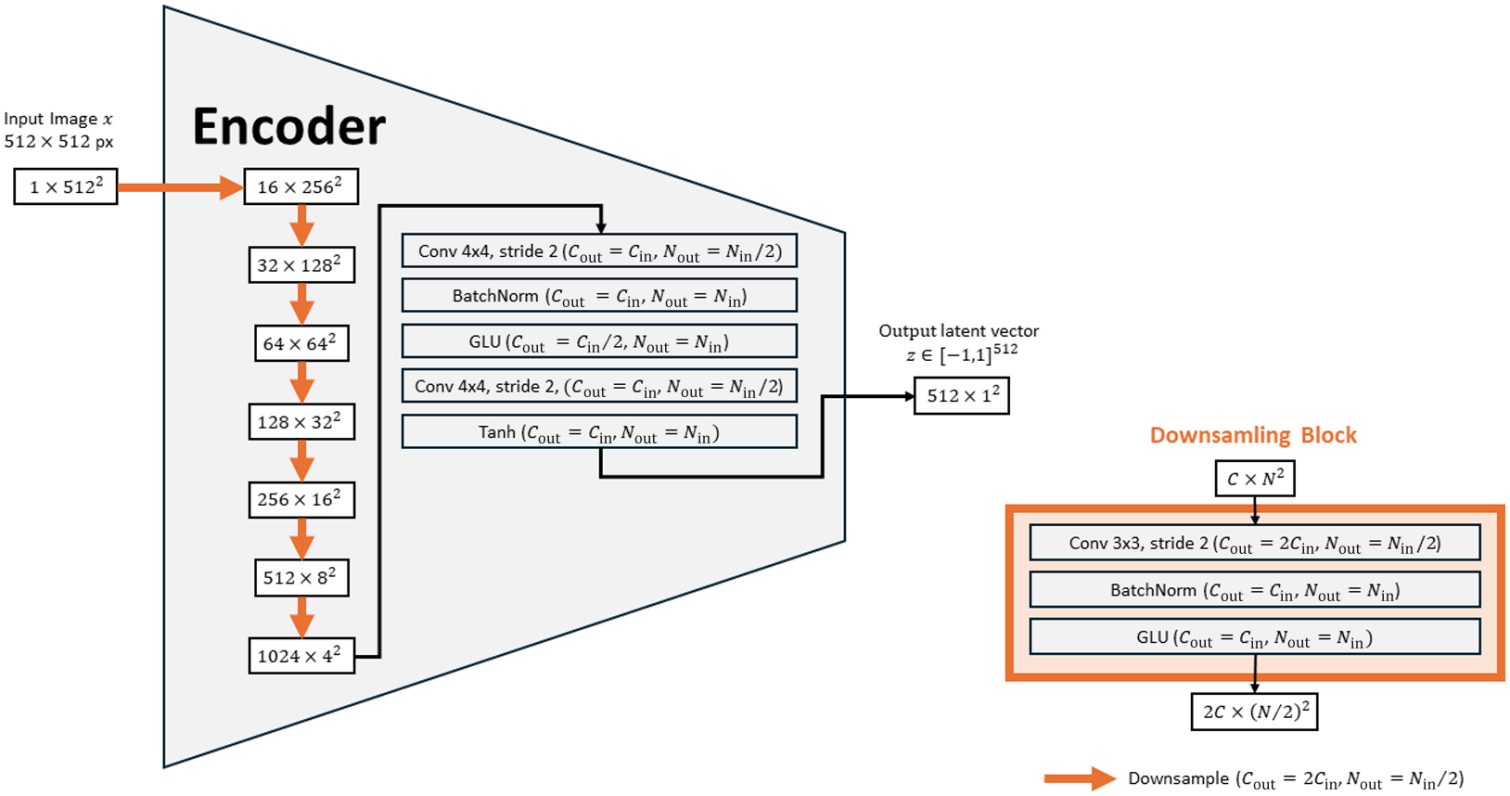
Schematic representation of our encoder architecture. The orange arrows represent a downsampling block that is shown on the right. C, channels; N, image dimension.

To reconstruct a normal representation of a test image, we need to encode the image x by a latent vector z, such that G(z)≈x, i.e., the generated image G(z) is similar to the input image x. Therefore, we train an encoder network E to obtain z from x. For the encoder training [Fig. 1(c)], the parameters of the generator remain fixed, and the discriminator is not used. We train the encoder by minimizing the encoder loss

$$L_{E}≔E_{x∼I_{\text{real}}}[w_{1}\ell_{\text{percept}}(x,G(E(x)))+w_{2}\ell_{\mathrm{MSE}}(x,G(E(x)))],$$ (3)

with so-called loss $\ell_{\text{percept}}$,^19^ mean squared error loss $\ell_{\mathrm{MSE}}$, and empirically chosen fixed weights $w_{1}=1$, $w_{2}=10$ balancing both loss terms. We use the same training dataset of 1180 normal brain CT slices as for the GAN training. In addition, we augment the images during training, such that random rectangular parts of the image are erased, meaning that the image in this area is set to the background value with an empirically chosen probability of 0.25. This should prevent the encoder from learning the identity function. The network is trained for 20,000 steps. The encoder architecture consists of eight down-sampling blocks that each include 2D convolution, batch normalization, and gated linear unit activation layers. A schematic representation of the encoder architecture is shown in Fig. 2.

#### 2.2.3 Image restoration: optimization of z

As an alternative to training an encoder and using the trained network during inference, we compute a solution z to the minimization problem

$$\underset{-1\leq z\leq 1}{\min}\;w_{1}\ell_{\text{percept}}(x,G(z))+w_{2}\ell_{\mathrm{MSE}}(x,G(z)),$$ (4)

with the loss $\ell_{\text{percept}}$,^19^ mean squared error loss $\ell_{\mathrm{MSE}}$, and empirically chosen fixed weights $w_{1}=1$, $w_{2}=10$ balancing both loss terms. We compute a numerical solution through iterative optimization of the objective function w.r.t. to the latent variable $z\in R^{512}$ starting from the initial guess $z=\overset{\to }{0}$. We used Adam optimizer, and z is clipped component-wise to the range [−1,1] at each step. The iteration is stopped after a fixed number of steps (8000), taking around 110 s. So, in this method, we do not train a network, but for each image, we iteratively compute our own individually optimized solution for inference.

#### 2.2.4 Obtaining anomaly maps

After training the networks, we are able to reconstruct a healthy version of a given input image using our iterative encoding method or the encoder, resulting in image pairs of the input image x and a normal representation x^=G(z). We then obtain a difference map $D=\rho (x-\hat{x})$ with a residual function ρ:R→R, e.g., ρ=|·| for detecting any change, which results in D=|x−x^|, or if we are trying to detect certain abnormalities that appear brighter than normal tissue, such as hemorrhages, we could choose ρ=max(·,0), which results in $D=\max(x-\hat{x},0)$.

Finally, we use a threshold to generate a binary anomaly mask. The mask is refined by applying a morphological closing operation and using a network ensemble. This means that we train our GAN three times with the same settings and thus also get three binary maps after reconstruction, which are then combined by a majority vote. By doing so, we can neglect regions that are falsely segmented as anomalies because of missing details in a single reconstruction.

### 2.3 Evaluation

We evaluate our method with two datasets. The first set includes 30 images with hemorrhages, whereas the second additionally contains 23 images with brain tumors. We show receiver operating characteristics (ROC) curves plotting pixel-wise false-positive rate against true-positive rate for different thresholds (see Fig. 7), and we report boxplots showing Dice overlap (Fig. 6). Other segmentation metrics such as the Haustorff distance would not provide meaningful results in our scenario, as there are often very small false-positive areas in the generated anomaly masks. Furthermore, we evaluate the realism of the images generated by our GAN through a visual Turing test.^21^^,^^22^ For this purpose, we mixed randomly generated and real images and asked radiologists and two medical imaging scientists to classify which images are generated and which are not.

## 3 Experiments and Results

For generating normal CT scans, we empirically set the dimension of the latent space to 512, i.e., we consider vectors $z\in [-1,1{]}^{512}$. Smaller dimensions led to decreased quality of the generated images showing repetitive image artifacts. Larger latent vectors did not further improve the results. As the method relies on the capability of the model to generate data that follow the distribution of normal healthy images, it is of interest to use a good generator. To give a quantitative analysis of the generated normal CT scans, we compute the unbiased Fréchet inception distance.^23^ Comparing the generated samples from the GAN, we obtain FID=19.01 for real healthy examples, whereas FID=30.48 for real disease images. Thus, our generated images are more similar to healthy images.

We evaluate the quality of the GAN images through a visual Turing test, where we randomly shuffled 25 real CT images and 25 CT images generated by our GAN, and two experienced radiologists and two experienced scientists from our medical imaging community evaluate which images were generated images. The results are summarized in Table 2. The radiologists rated the image quality as very good and were unable to correctly classify ∼20% of the images. Our medical imaging colleagues were unable to distinguish between fake and real images.

**Table 2:** Turing test results.

Reader	Scientists			Radiologists		
	A	B	(A+B)/2	C	D	(C+D)/2
TP (out of 25)	7	11	9	22	19	21.5
TN (out of 25)	13	12	12.5	17	21	19
Recall TP / (TP + FN)	0.37	0.46	0.42	0.73	0.83	0.78
Precision TP / (TP + FP)	0.28	0.44	0.36	0.88	0.76	0.82
Accuracy (TP + TN) / (P + N)	0.40	0.46	0.43	0.78	0.80	0.79

TP, number of correctly classified generated images; TN, number of correctly classified real images.

**Fig. 3:**
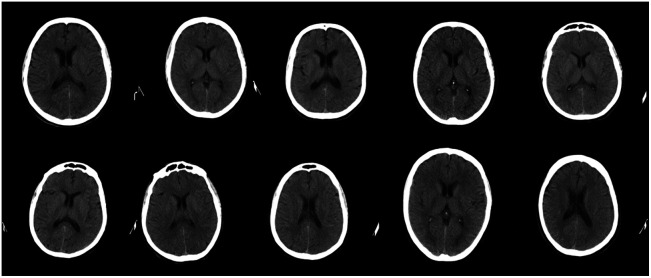
Exemplary output images from our trained generator from randomly sampled latent vectors.

In addition, qualitative examples of the generated images are shown in Fig. 3. It is visible that a variety of ventricle and skull shapes are represented by the generated images. Brain tissue details such as sulci or center line are recognizable, which lets the images appear realistic.

To obtain a further quantitative value for the reconstruction quality, we calculated the root mean square error (RMSE) between the input and output images for each of the 100 real CT slices with and without anomalies. For this purpose, the intensity range of the images was normalized to the interval [0,1]. Although we obtain a mean and standard deviation of the absolute difference of 0.032±0.081 and an RMSE of 0.089 for the image pairs with anomalies, the mean ± standard deviation and the RMSE for normal image pairs are only 0.024±0.070 and 0.076, respectively. Moreover, the 90% percentile of the pixel difference was 0.074 for normal images and 0.106 for images with abnormalities. To summarize, we achieve good reconstruction quality for healthy images. The measured reconstruction quality of the healthy images is significantly better compared with images with abnormalities, which is due to the fact that abnormal areas are poorly or “healthy” reconstructed. In addition, the quality of healthy image reconstruction is sufficient so that the images can be differentiated into two classes (normal and abnormal), as in Walluscheck et al.^18^

**Fig. 4:**
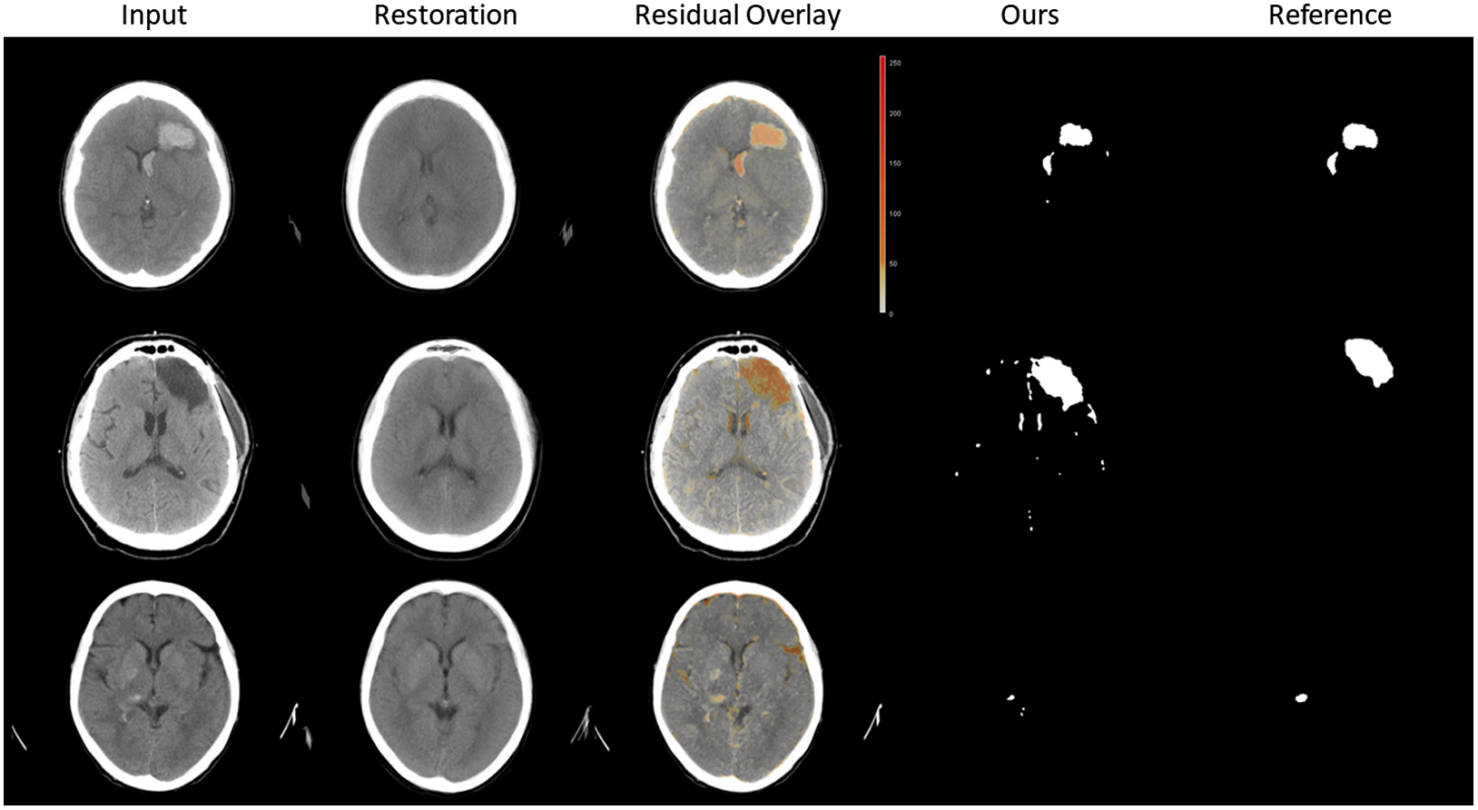
Exemplary results on images from our hemorrhage (rows 1 and 3) and tumor test set (row 2). Our segmentation is obtained from the residual by thresholding with the best value obtained from the ROC analysis on our validation set. Reference: manual segmentations for hemorrhages and tumors.

A qualitative impression of our results for three test images is given in Fig. 4. The shown reconstructions are average images of the reconstructions of three GAN models and therefore appear blurrier than the individual reconstructed images. The shown residual is color-coded to make it visible in the overlay. Stronger deviations appear in a darker orange color.

**Fig. 5:**
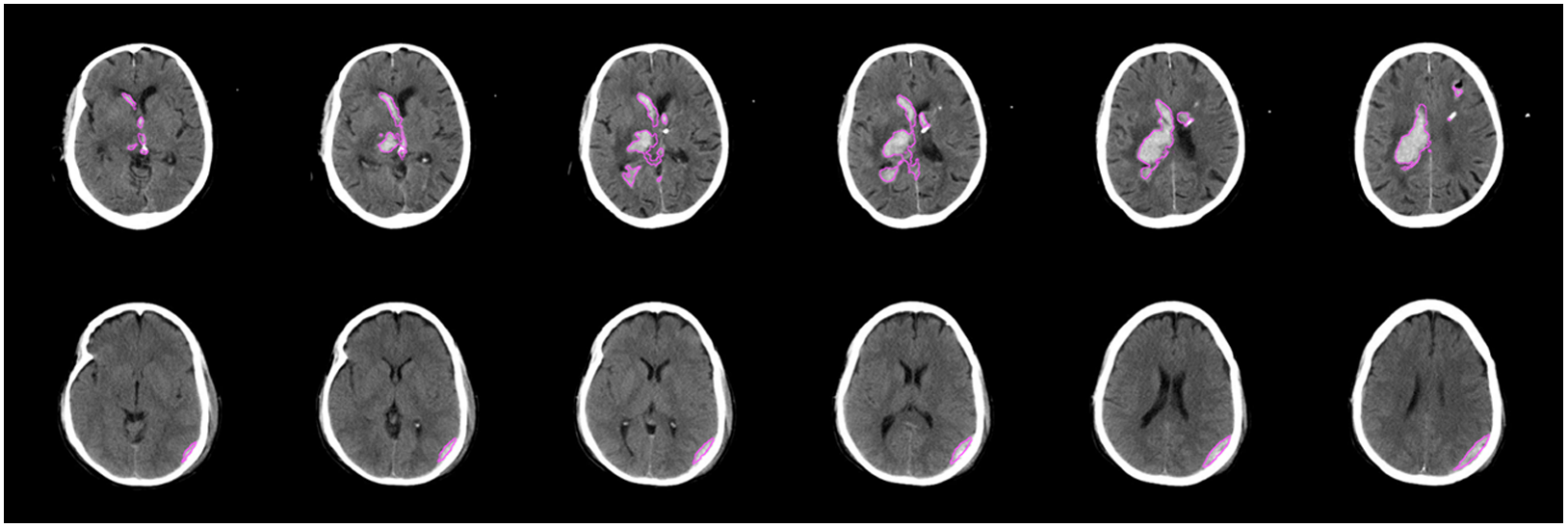
Exemplary segmentation results on subsequent axial slices with hemorrhages for two patients.

We also test the performance of our method on CT volumes. Figure 5 shows the segmentation overlay on several consecutive axial slices for two test volumes. We observe that using our method on 2D slices of CT scans leads to sensible results when applied to subsequent slices. As done here, a connected component analysis on the results of multiple slices can further reduce small false-positive findings.

### 3.1 Hemorrhage Dataset

**Fig. 6:**
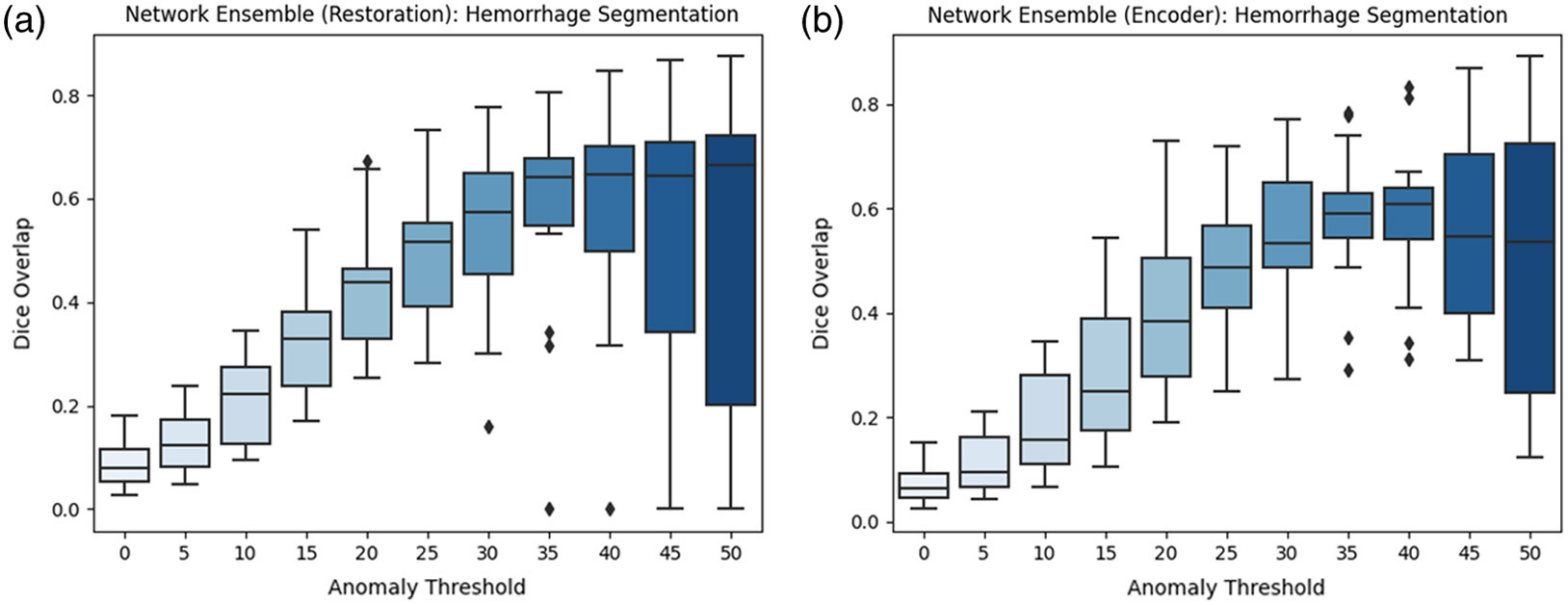
Dice overlap on hemorrhage data from the validation set for different anomaly thresholds. (a) Our model with restoration. (b) Our model with encoder.

When testing on hemorrhage data, only positive values are considered because hemorrhages appear brighter than healthy tissue. In Fig. 6, we present the Dice overlap for increasing the anomaly threshold for our model with encoder and with restoration on the validation set (15 images). We observe that our results highly depend on the threshold. After choosing a threshold on the validation set (t=36), the network with restoration achieved a median Dice overlap of 0.71 (mean Dice 0.62±0.24) on the test dataset (15 images). The Dice for the version with an encoder is slightly lower with a median of 0.66 (mean Dice 0.61±0.25) leading to a p-value of 0.96 in a statistical Wilcoxon test. We detect 91.7% of the reference lesions. A lesion is counted as detected when the Dice is >0.1 for both network versions. We also tested the supervised hemorrhage segmentation tool BlastCT^20^ on our test data and obtained a median Dice score of 0.73. The difference in our results is not statistically significant (p-value >0.5). Therefore, our methods achieve similar results as this supervised approach.

### 3.2 Combined Dataset

As our approach is not trained to detect a specific type of lesion, it can be used on data, including multiple anomalies. When testing on data with tumors and hemorrhages, we do not consider any prior knowledge of image values. Thus, the anomaly threshold is applied to the absolute values of the difference image. First, we determine a threshold on the validation set (27 images) and use this operating point to report results on our test data (26 images).

The best result is obtained with a median Dice overlap of 0.43 (mean Dice 0.40±0.18) for the restoration and 0.30 (mean Dice 0.37±0.19) for the encoder network version (Table 3). A statistical test on both results leads to a p-value of 0.02 showing statistical significance. We observe that, overall, the Dice values are lower than the previously presented results for the hemorrhage dataset. However, we would like to emphasize that we did not develop a method for the exclusive detection of hemorrhage and therefore include these data in our evaluation. Furthermore, we achieved an accuracy of 92.9% on the tumor data in our test set, which means that our method is suitable for application to multiple types of abnormalities.

**Fig. 7:**
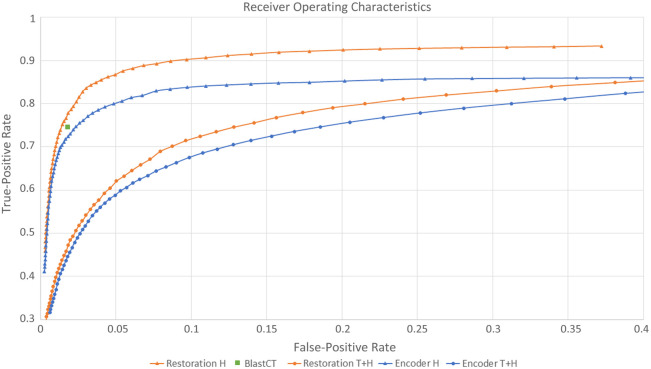
ROC curves for two test datasets (H, hemorrhage; T+H, tumor and hemorrhage) for two network versions (restoration and encoder). The result for the hemorrhage detection tool BlastCT^20^ is marked as a point.

In Fig. 7, we show the ROC curves for the model with encoder and restoration for two test sets. We see that the restoration method performs better than the encoder model. Also, the performance for both models is better on the hemorrhage test set than on the combined test set. In addition, we mark the performance of the BlastCT tool^20^ (tested on hemorrhage data) as a point in the graph and see that it is similar to our models.

**Table 3:** Summary of the anomaly segmentation for two network variants on two test sets showing the mean Dice values with standard deviation.

Test set	Dice	
	Restoration	Encoder
Hemorrhage	0.62±0.24	0.61±0.25
Hemorrhage and tumors	0.40±0.18	0.37±0.19

### 3.3 Comparison with Other Models

A natural candidate for comparison to our method would be the work of Lee et al.,^13^ which has been proposed for emergency triage in brain CT scans. Their results are of high quality and very promising. We applied the available trained network to our test data but obtained unsatisfactory reconstruction results that showed strong deviations from our input images. Therefore, we refrain from further quantitative analysis for comparison here.

In addition, we compare our model with two state-of-the-art models, namely, an autoencoder^24^ and a diffusion model called “AnoDDPM,”^10^ that were both proposed for anomaly detection in brain MR images. We trained both methods on our training dataset and applied them to our hemorrhage test data (15 images).

**Fig. 8:**
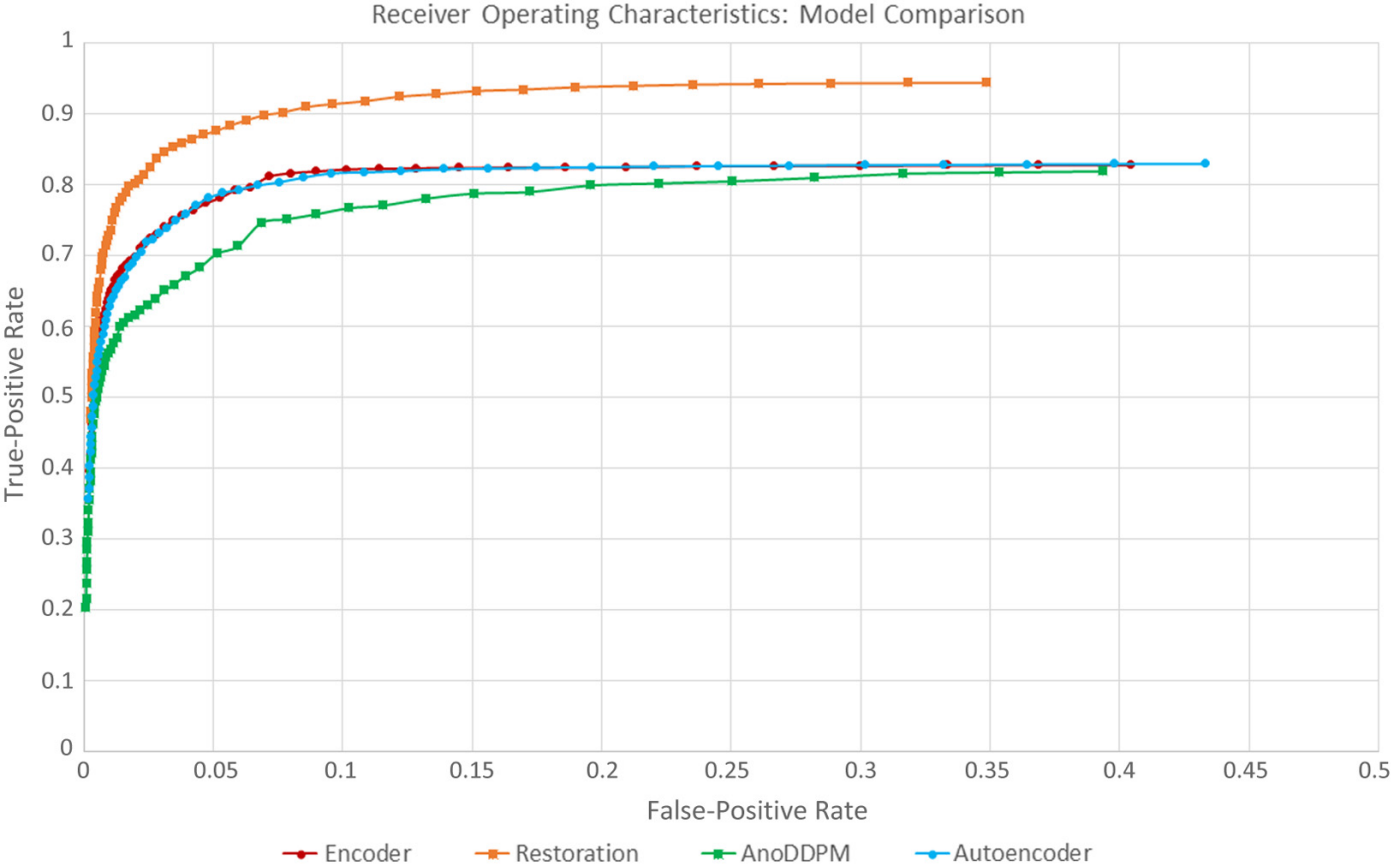
ROC curves for two recently published models and our two model versions on our hemorrhage test dataset.

**Fig. 9:**
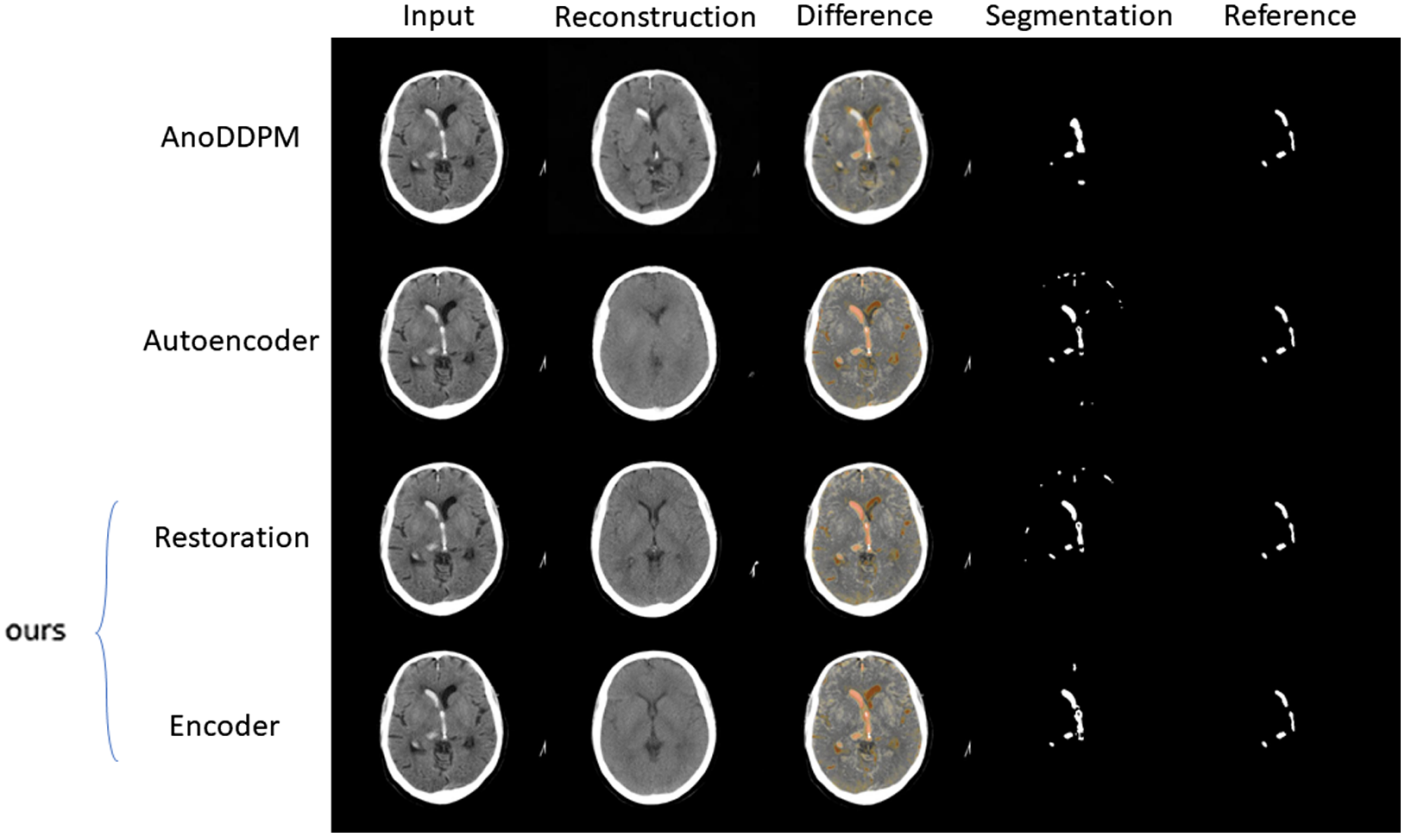
Qualitative comparison of the reconstruction performance for the diffusion model AnoDDPM, an autoencoder, and our two network versions on a hemorrhage test case.

Figure 8 shows ROC curves for the autoencoder, diffusion model, and our two network versions on our hemorrhage test dataset. It shows that our method performs slightly better. To get a visual impression of the performance difference, we show the results for all four models on a disease test case with prominent hemorrhages in Fig. 9.

We note that the hemorrhage within a ventricle is still visible in the reconstruction of the AnoDDPM. We found that the noise distribution used for the diffusion model has a strong influence on what kind of abnormalities are removed in the reconstruction. This could be a disadvantage as it cannot be ensured that the reconstruction always represents normal anatomy. We note that the hemorrhage within a ventricle is still visible in the reconstruction of the AnoDDPM. We found that the noise distribution used for the diffusion model has a strong influence on what kind of abnormalities are removed in the reconstruction. This could be a disadvantage as it cannot be ensured that the reconstruction always represents normal anatomy. The image reconstructed by the autoencoder does not show details inside the brain tissue and is generally blurred. In this example, we see that our model provides images with normal anatomy, even for this example image that shows a brain heavily affected by bleeding. Our network produces sharp and detailed images that contain no abnormalities.

## 4 Discussion and Conclusion

We present a method for detecting nonspecific abnormalities in brain CT scans that does not require annotated data. Unlike many GAN approaches, the architecture we have chosen requires only a very small amount of training data and produces high-resolution images that are less blurred than the VAE or AE methods. Instead of learning to detect specific abnormalities, we learn normal representations of brain CT scans and detect the deviations from normal in unseen images. We train with healthy 2D CT slices only and show that we can detect and localize hemorrhages and tumors. When testing our unsupervised approach with hemorrhage data, we obtain good results comparable to a recent supervised CT segmentation tool (Monteiro et al.^20^) for hemorrhages. Thus, we show that our approach can localize lesions that were not specified during training. Our network is not susceptible to the size of the anomalies as we compare the images per pixel and do not filter or constrain the output according to the residual size.

By training a GAN to produce normal images in the first step, we ensure that we only obtain images that show normal anatomy, regardless of the type or severity of the abnormality in the input image. At the same time, our reconstructions have good image contrast and show more details of the brain tissue than comparable autoencoder approaches.

For our model, we compare two approaches to image reconstruction, namely, training an encoder and iterative optimization for image restoration. We find that both methods produce very similar reconstructions. In the quantitative evaluation, the restoration method seems to perform slightly better, but the difference is not statistically significant. Also, the restoration model requires 110 s to reconstruct a 2D image, whereas the encoder model takes only around 0.02 s, which could be advantageous in an application.

As our method is based on grayscale comparisons, the performance is limited by the gray value deviation of the lesion compared with the healthy tissue. Very subtle lesions are therefore likely to be missed. However, this is a general observation that affects any approach based on detecting lesions by analyzing image value intensities and also includes manual detection by radiologists. However, a limitation of our work is the limited training dataset of 1180 images and the small test dataset of 26 images. It would be a great future addition to further test the model on a larger amount of data if more images were available.

We have shown that our method produces consistent results when applied to consecutive axial slices and is therefore suitable to locate lesions in CT volumes.

By learning a representation of normal CT scans, we can reconstruct the overall anatomy of a healthy brain. Thus, structural changes such as ventricular deformations are also corrected in our reconstruction. In the future, detection of such changes may also be possible with our method. Anatomical details such as the exact location of the sulci of a particular patient are not optimally reconstructed. Therefore, these differences between input and reconstruction are currently detected as anomalies in some cases, as can also be seen in Fig. 4 (small extra areas in the segmentation mask). In future work, we would like to investigate how to distinguish between these “irrelevant” differences and true anomalous differences. Possible research could include developing learning-based methods to obtain anomaly maps from the reconstructed images rather than computing the difference image.

## Data Availability

In this study, we used two publicly available datasets. The RSNA data that we used for training are available from Ref. 14. For testing, we utilized a glioma dataset that is available from the Cancer Imaging Archive.^15^ Due to the research being part of a running project funded by the Federal Ministry of Education and Research of Germany (BMBF), the code developed for this work cannot be made publicly available.
